# Medial unicompartmental knee arthroplasty in the ACL-deficient knee

**DOI:** 10.1007/s10195-016-0402-2

**Published:** 2016-05-09

**Authors:** Francesco Mancuso, Christopher A. Dodd, David W. Murray, Hemant Pandit

**Affiliations:** 1 Nuffield Department of Orthopaedics, Rheumatology and Musculoskeletal Sciences (NDORMS), University of Oxford, Oxford, UK; 2 Orthopaedics and Traumatology Unit, “Santa Maria della Misericordia” University Hospital, Udine, Piazzale Santa Maria della Misericordia 15, 33100 Udine, UD Italy

**Keywords:** Medial compartment osteoarthritis, Anterior cruciate ligament deficiency, Anterior cruciate ligament reconstruction, Unicompartmental knee arthroplasty

## Abstract

Symptomatic osteoarthritis (OA) of the knee develops often in association with anterior cruciate ligament (ACL) deficiency. Two distinct pathologies should be recognised while considering treatment options in patients with end-stage medial compartment OA and ACL deficiency. Patients with primary ACL deficiency (usually traumatic ACL rupture) can develop secondary OA (typically presenting with symptoms of instability and pain) and these patients are typically young and active. Patients with primary end stage medial compartment OA can develop secondary ACL deficiency (usually degenerate ACL rupture) and these patients tend to be older. Treatment options in either of these patient groups include arthroscopic debridement, reconstruction of the ACL, high tibial osteotomy (HTO) with or without ACL reconstruction, unicompartmental knee arthroplasty (UKA) and total knee arthroplasty (TKA). General opinion is that a functionally intact ACL is a fundamental prerequisite to perform a UKA. This is because previous reports showed higher failure rates when ACL was deficient, probably secondary to wear and tibial loosening. Nevertheless in some cases of ACL deficiency with end-stage medial compartment OA, UKA has been performed in isolation and recent papers confirm good short- to mid-term outcome without increased risk of implant failure. Shorter hospital stay, fewer blood transfusions, faster recovery and significantly lower risk of developing major complications like death, myocardial infarction, stroke, deep vein thrombosis (as compared to TKA) make the UKA an attractive option, especially in the older patients. On the other hand, younger patients with higher functional demands are likely to benefit from a simultaneous or staged ACL reconstruction in addition to UKA to regain knee stability. These procedures tend to be technically demanding. The main aim of this review was to provide a synopsis of the existing literature and outline an evidence-based treatment algorithm.

## Introduction

Few rules are known in medicine, but one of these assumes that unicompartmental knee arthroplasty (UKA) for medial osteoarthritis (MOA) is contraindicated if anterior cruciate ligament (ACL) is functionally deficient.

This has been generally accepted since the first reports highlighted a higher incidence of complications, in terms of tibial loosening and higher revision rate, when UKA were performed in ACL-deficient knees [9, 11].

**Fig. 1:**
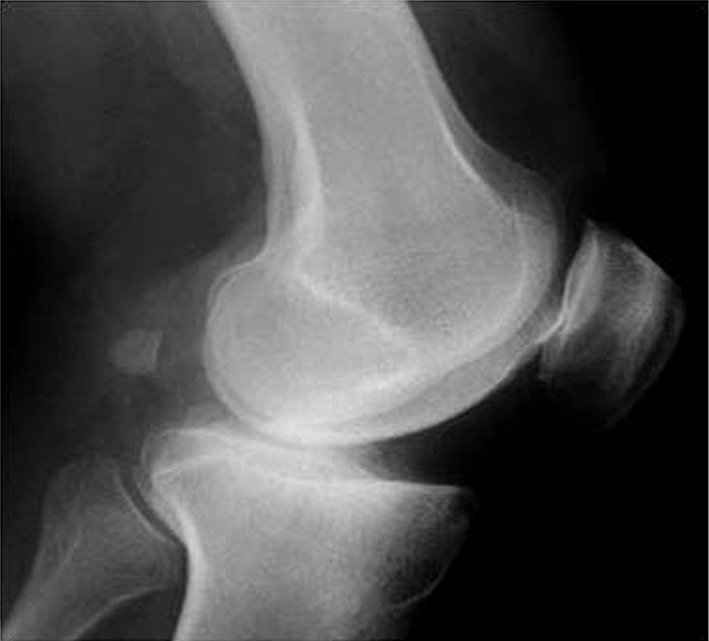
Lateral X-ray showing posterior wear of the tibial plateau in an anterior cruciate ligament (ACL)-deficient knee

Primary MOA in an ACL-intact knee usually involves the antero-medial aspect of the inner compartment and is therefore called antero-medial osteoarthritis. The preserved postero-medial compartment maintains a functional medial collateral ligament (MCL) [25] as every time the knee flexes, the femur rides out of the tibial defect allowing the MCL to regain its normal length. In such knees, with the passage of time, if the antero-medial OA is not treated, the wear patch on the medial tibial plateau extends posteriorly, the ACL progressively becomes damaged, typically from notch osteophytes, and eventually ruptures. These patients typically exhibit a more extensive wear pattern involving also the posterior aspect of the medial compartment [27]. In patients with primary ACL damage and secondary knee OA due to repeated episodes of anterior subluxation of the tibia in respect of the femur, the tibial wear patch is typically postero-medial, allowing normal antero-medial cartilage (Fig. 1). Every time the patient moves his/her knee, the femur rides free of the defect and corrects the varus deformity, thereby maintaining the normal length and functionality of the MCL.

Typical indications for UKA are a stable knee, functionally intact lateral and femoro-patellar compartments, correctable (intra-articular) varus deformity, less than 10°–15° of fixed flexion deformity, and flexion beyond 100°. Outside these indications, typically a patient should be offered a total knee arthroplasty (TKA). If usual indications are applied, UKA guarantees several advantages over TKA, in terms of better range of motion, less soft tissue damage allowing early and rapid recovery, preservation of bone stock, minimal blood loss, lower complication rates (including significantly reduced risks of stroke, heart attack, death or venous thromboembolism) and preservation of normal kinematic function. Furthermore, with an intact ACL, many series have shown that Oxford medial unicompartmental knee arthroplasty achieved survival rates of more than 90 % at 10 and 15 years, irrespective of patient’s age or activity level. In addition, the wear rates of an Oxford UKA are significantly lower than those of fixed bearing UKA or TKA due to its unique design characteristics of a fully congruous mobile bearing UKA maximising the contact area and minimising the contact stresses throughout the arc of knee flexion [19, 26].

These are the key reasons for UKA to be a preferred and appealing treatment option for either young and/or active patient or more elderly patient with significant co-morbidities even if ACL is deficient. TKA may represent a suboptimal option in terms of implant longevity in the former group and in terms of comorbidities in the latter.

## Indications

**Fig. 2:**
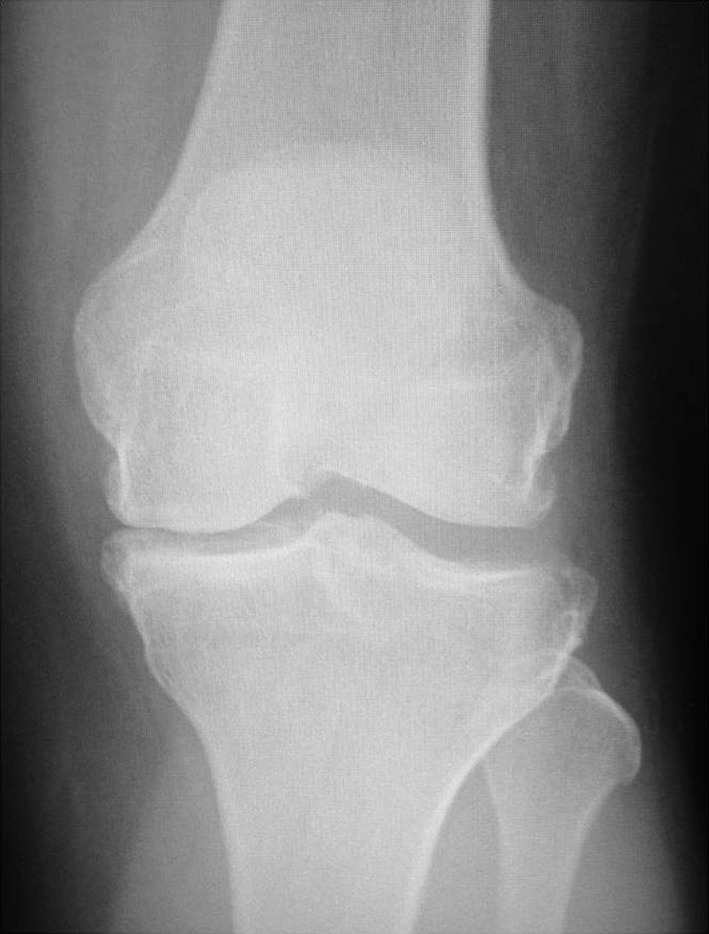
End-stage medial compartment osteoarthritis (MOA)

Indications for UKA are based primarily on patho-anatomy rather than patient characteristics. Any patient with anteromedial OA and bone-on-bone arthritis with intact lateral cartilage (Fig. 2) and correctable varus deformity are ideal candidates for UKA, provided the ACL is intact or is reconstructed. If partial thickness lesions are present, especially in a malaligned knee, high tibial osteotomy (HTO) may become the preferred treatment of choice.

However, when approaching a patient with MOA in ACL-deficient knees, the main features to take into account are patient’s biological age, functional demands and primary symptom.

Age and functional activity play a significant role in our decision regarding whether to reconstruct the ACL or not. Elderly patients with lower functional requests, may benefit from the UKA without ACL reconstruction. On the other hand, in younger patients with isolated MOA, an ACL reconstruction, regaining stability in their knee, is preferred.

**Fig. 3:**
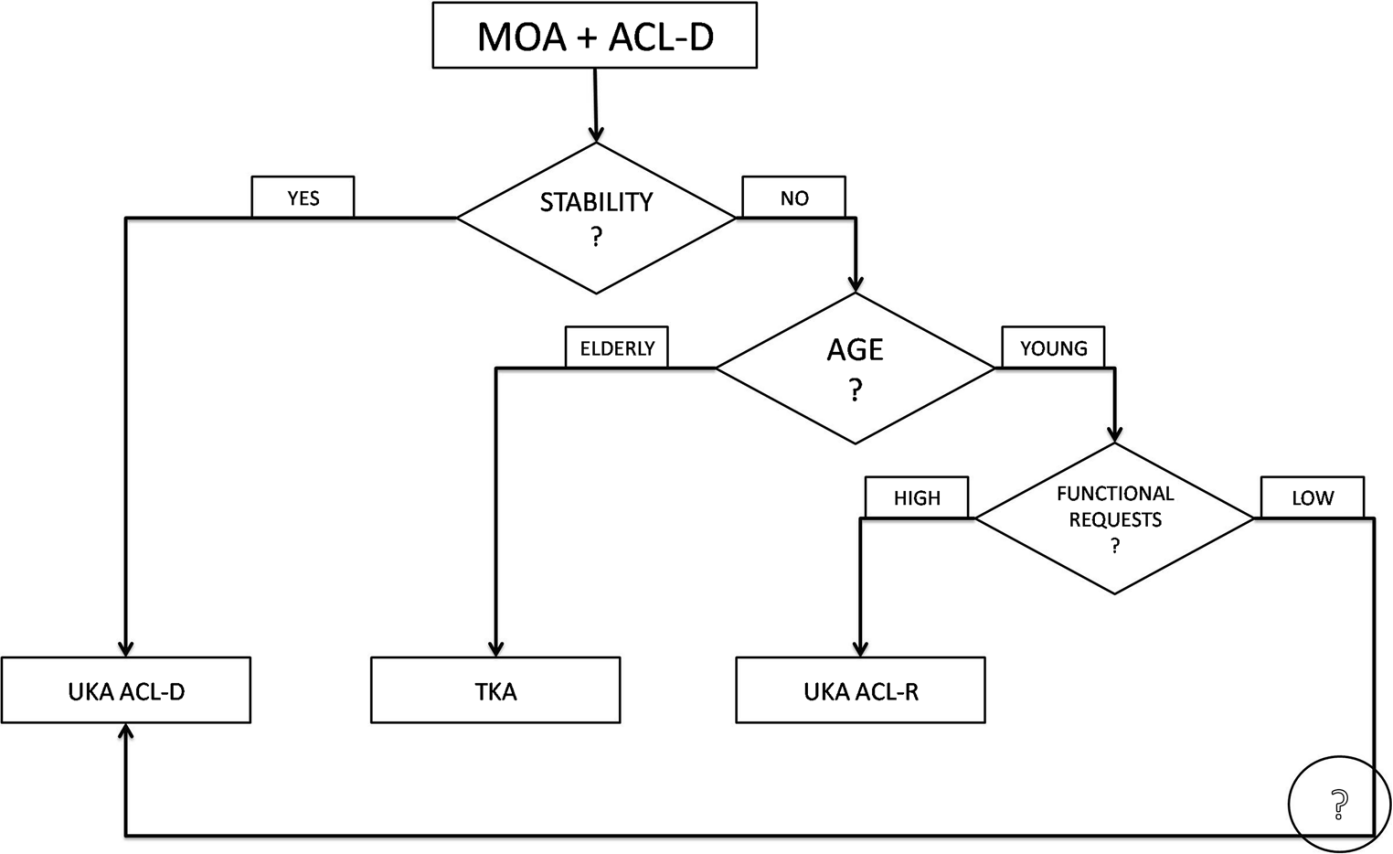
Suggested treatment algorithm for medial osteoarthritis (MOA) and ACL-deficient knees

In the subjective evaluation of these patients, mechanical pain is usually present due to the MOA, eventually associated with a swollen knee. On the other hand, instability, even if ACL is deficient, may not be referred as a main symptom, probably because of the muscular status, the functional requests or the presence of posterior osteophytes [18] and capsule stiffness, which contribute to knee stability. In those patients, ACL reconstruction may not be performed to avoid further surgical steps and increasing arthrofibrosis risk. Considering the ACL-deficient knee, it is important to identify those without functional impairment and instability, known as “copers”, who are able to resume pre-injury activity level without the need for ACL reconstruction [14]. According to those assumptions a treatment algorithm may be drawn (Fig. 3).

Lower limb alignment is an important factor to consider in the assessment of a painful MOA knee, independently of ACL status. In the presence of extra-articular deformity, and initial-to-moderate OA, HTO would be the treatment of choice to correct the varus malalignment, thus restoring a neutral mechanical axis and reducing pressures on cartilage defects [12]. In contrast, a well aligned knee is better approached with a UKA, because its main aim is to restore the ligament to normal rather than to correct limb alignment without altering the physiological joint line [10].

If these considerations are accepted as a general rule, challenges may arise in the management of patients with malalignment and advanced disease, and patients with normal alignment but early partial thickness disease. The former group may benefit from a TKA rather than an HTO as their results in advanced OA are known to be poor [7]. In young patients, however, HTO is still an option to delay more invasive procedures like arthroplasties, even if symptoms relief may be partial. On the other hand, patients with good alignment and partial thickness disease should be not approached with an HTO to avoid unphysiological alteration of the joint line. UKA would also not be appropriate because, in the setting of partial thickness disease, results are worse than with bone on bone arthritis, and post-operative outcomes are less predictable [22].

## Technical features

Nowadays UKA is a well standardised operation but performing it in ACL-deficient knees adds uncertainty about whether the ACL should be reconstructed or not.

**Fig. 4:**
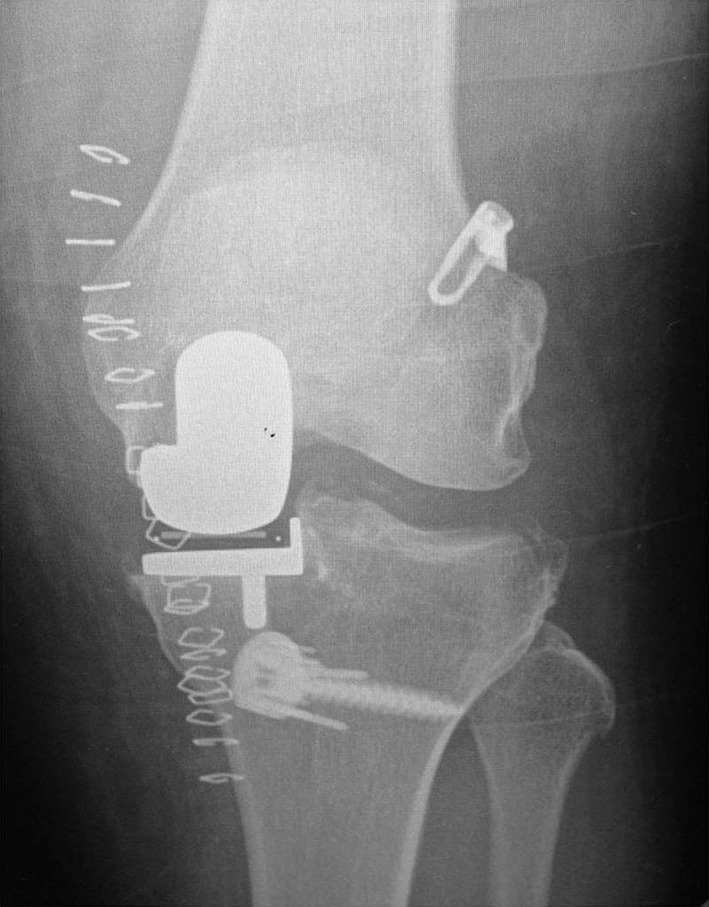
Post-operative X-ray of a combined unicompartmental knee arthroplasty (UKA) and ACL-reconstruction

ACL reconstruction can be performed simultaneously or in a staged procedure. Combined UKA and ACL reconstruction (Fig. 4) becomes a longer and more technical demanding procedure but avoids the need for a re-operation, with one more anaesthesia, longer recovery time and higher social costs. A staged procedure starting with ACL reconstruction may be indicated if instability is the main symptom, proceeding with the UKA only if pain arises later.

**Fig. 5:**
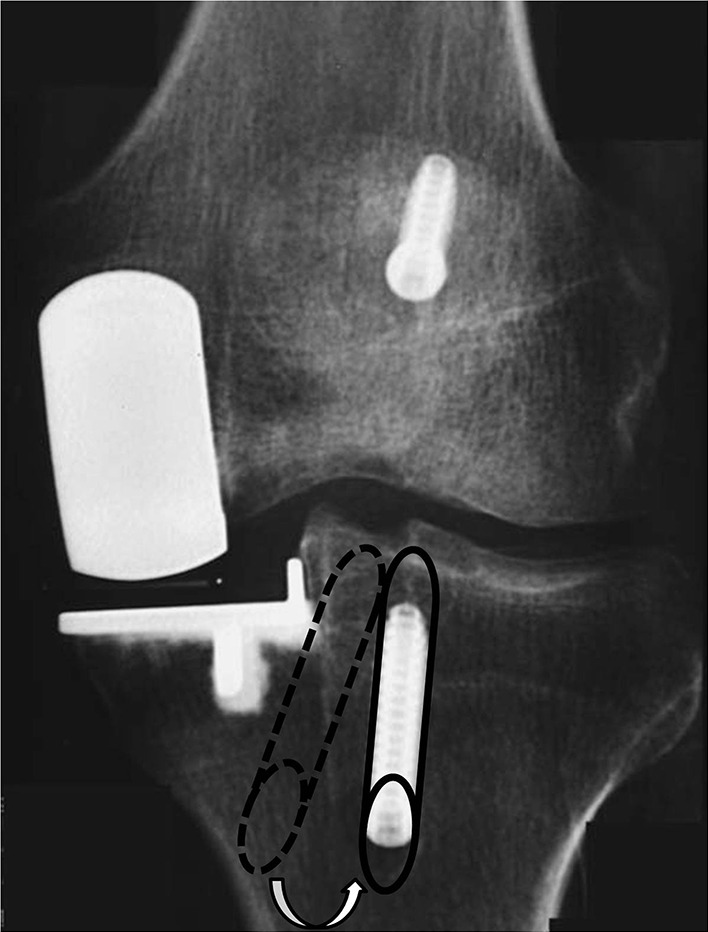
Lateralised and verticalised tibial tunnel in combined UKA and ACL-reconstruction

The surgical technique has been described by different authors [6, 21, 31, 33]. One key technical aspect is to avoid impingement of the graft tunnel on the tibial component of the UKA [15], and a second key aspect is to tension the graft properly. In addition, it is also possible that one may weaken the tibial plateau, leading to an additional risk of tibial fracture. Therefore, the advice is to perform the tibial tunnel slightly more laterally than usual [31] and/or in a more vertical direction to reduce the medial stress/impingement (Fig. 5). If cementless implants are used, the tibial tunnel should be drilled after positioning of the tibial component to lower the risk of fracture during tibial implant application. There is no clear evidence for this suggestion although it is intuitive to do so. After drilling the femoral tunnel and fixing the femoral end of the ACL graft, one can complete the implantation of the UKA. Finally, the tibial end of the graft can be fixed at the end of the procedure to achieve the right tension.

The choice of graft is not clearly stated in the literature [21]. Mainly bone-patellar tendon-bone (BPTB) and hamstring autografts are used but other options described include the use of allografts and synthetic implants. For the one-stage procedure, our opinion is to favour a bone-tendon-bone graft rather than a hamstring graft because it provides stronger initial fixation (bone to bone rather than bone to tendon), and the tibial tunnel can be drilled through the donor site in the tibial tubercle and so slightly lateralised, as previously mentioned. The medial third of the patellar tendon may be harvested through the traditional UKA approach, thus reducing the operative morbidity of the traditional middle third, which may lead to devascularisation of the remaining medial portion of the patellar tendon [15].

Tibial slope modification has been reported to play a role in ACL strain and knee stability. Opening and closing wedge osteotomies, increasing [3, 17] and decreasing [13] the posterior slope, respectively, have an effect on knee stability if performed in ACL-deficient patients. In the same way, tibial tray slope may be modified in UKA to reduce anterior tibial translation in ACL-deficient knees, as reported by Suero et al. [28] in a cadaveric study with fixed bearing UKA in non-weight bearing conditions. They showed an anterior tibial translation of about 5 mm, close to the intact knee, during a Lachman test with an 8° levelling of the posterior tibial slope. However, rotational stability during a pivot shift test was not influenced by slope modifications [28]. The role of tibial slope was confirmed also in a retrospective clinical paper by Hernigou and Deschamps [11]. This latter study found an increase in aseptic loosening rate if slope was more than 7° with a fixed bearing UKA, thus recommending not to exceed this limit [11].

The choice between mobile and fixed bearing depends partially on the surgeon’s preference. The potential for accelerated polyethylene wear, when performing an ACL-deficient UKA, is one of the main concerns that frighten surgeons. Fixed bearings are usually flatter, allowing a sliding motion of the femoral condyle. On the other hand, mobile bearings present a sliding motion on their inferior aspect over the tibial tray while a rolling motion of the femoral component is expected on the congruent superior surface. In laboratory evaluation, Blunn et al. [1] found a dramatically increased polyethylene wear with cyclic sliding compared with compression or rolling because of increased subsurface shear stresses, concluding that low-conformity components (i.e. fixed bearings) inserted with high ligamentous laxity are susceptible to antero-posterior sliding and hence high wear [1].

Table 1 reports survival rates of UKA by bearing type, obtained by pooling data from published papers about UKA in ACL-deficient knees. Although clinical outcomes are fairly similar, ACL reconstruction with fixed bearing seems to be the best choice in terms of survival rates, failures and revisions/100 observed years, although it is impossible to draw definitive conclusions due to the relatively short follow up period (65 months) and the small size of the population involved (262 knees). One can expect that mobile bearing is at risk of instability or dislocation but, according to these data, only two cases are reported, one each in the ACL-deficient and ACL reconstructed groups.

**Table 1:** Survival rate data for fixed and mobile bearing unicompartmental knee arthroplasty (UKA) with or without anterior cruciate ligament (ACL) reconstruction

Group	Patients (*n*)	Mean age, years (range)	Mean follow up, months (range)	Raw survival rate (%)	Failures/100 observed years	Revisions/100 observed years
Mobile						
ACL-deficient	74	67 (54–77)	50	91	2.26	2.26
ACL-reconstructed	61	51 (36–71)	54	95	1.17	1.17
Total	135	60 (36–77)	52 (12–120)	92.8	1.77	1.77
Fixed						
ACL-deficient	80	66 (39–91)	102	85	1.77	1.77
ACL-reconstructed	47	49 (38–64)	40	100	0	0
Total	127	60 (38–91)	79 (9–264)	90.6	1.44	1.44

One more concern is the definition of intact ACL. Pre-operative assessment performed by clinical test and/or MRI study can under- or over-estimate the prevalence of ACL lesion. The presence of bone deformities, osteophytes or soft tissue contracture may alter the perception of antero-posterior laxity [2]. Our preferred option is intra-operative assessment under direct visualisation. We use a tendon hook and pass it around the native ACL and give a hard pull. If the ligament gets pulled off then clearly the ACL was deficient but if it does not then it is considered functionally intact. We have used this criterion for the last 35 years and have not found any reasons/evidence to change our practice. It is possible that an anatomically intact ACL has already lost its functional role due to degenerative changes in the microstructure, as has been reported by Trompeter et al. [32], who showed that greater than two out of three ACLs found to be macroscopically normal during TKA present moderate-to-severe disease at microscopic assessment.

## Results

### Biomechanical studies

An in vitro robotic studies by Suggs showed that knee stability is not altered by a medial fixed bearing UKA but they conclude that ACL is essential to avoid greater anterior tibial translation [29]. Their findings on similar ACL forces in the native knee and following UKA led to the conclusion that ACL also plays a role in the latter condition. Of course this is true assuming that UKA is well balanced with equal flexion and extension gaps, otherwise it may happen that ACL loses its physiological strain, thus rendering it non-functional. Citak et al. [5] demonstrated restored knee kinematics after performing a combined ACL reconstruction and fixed bearing UKA on cadaver specimens. In particular, there was no significant difference in lateral compartment translation during the Lachman and pivot shift tests between the ACL-intact UKA and the ACL-reconstructed UKA.

**Fig. 6:**
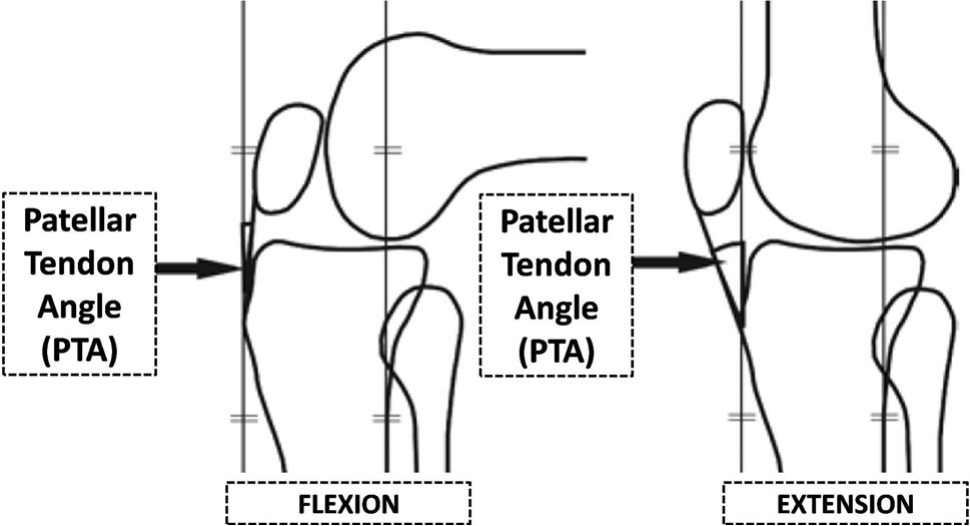
Patellar tendon angle (PTA) in knee flexion and extension

In vivo knee kinematics studies have also been performed, analyzing patellar tendon angle (PTA) in the sagittal plane (Fig. 6) as a marker of knee kinematics during high demand exercises between full extension and flexion [23]. PTA is a good measure of both patello-femoral and tibio-femoral joint kinematics, and is related to both the patella-femoral and the tibio-femoral contact forces. Major abnormalities in the PTA are likely to be a result of abnormalities in the relationship of the femur to the tibia. Anterior subluxation of the femur increases the angle, whereas posterior subluxation decreases it. Pandit et al. [23] found that normal kinematics is restored in vivo after ACL reconstruction in UKA, even if a slight anterior tibial (or posterior femoral, considering a closed chain exercise) displacement persists. This may determine similar components loading and, eventually, similar long-term survival. In ACL-deficient knees Pegg et al. [24] showed different knee kinematics between ACL-deficient and ACL-intact patients after UKA, particularly noticeable during the step-up between 30° and 60° of flexion, with a decrease in PTA in the ACL-deficient group. Overall, the kinematics of the ACL-deficient knees seemed to be more physiological than data reported for TKA, but not as close to healthy knees as ACL-reconstructed UKA knees.

### Clinical studies

Several clinical studies have reported UKA results in ACL-deficient patient, combined [6, 15, 31, 33, 34] or not [2, 8, 9, 11] with ACL reconstruction stage. Demographic data are reported in Table 2.

**Table 2:** ACL-deficient and ACL-reconstructed UKA demographic data and surgical details from the literature

Year	Authors	Journal	Initial cohort	Female (%)	Mean age, years (range)	Mean follow up, months (range)	Percentage follow up (%)	Bearing type	Single-stage approach (%)
ACL-deficient UKA									
1988	Goodfellow et al. [9]	JBJS Br	28	NR	70 (62.4–77.6)	36 (21–56)	28/28 (100)	Mobile	–
2004	Hernigou and Deschamps [11]	JBJS Am	18	NR	70 (43–83)	204 (180–264)	18/18 (100)	Fixed	–
2012	Boissonneault et al. [2]	KSSTA	46	23.8	65 (54–76)	58.8 (26.4–91.2)	46/46 (100)	Mobile	–
2013	Engh and Ammeen [8]	CORR	70	48.3	65 (39–91)	72 (34–120)	62/70 (88.6)	Fixed	–
Total	Four studies		162	38.2	66 (39–91)	77 (26.4–264)	154/162 (95.1)		–
ACL-reconstructed UKA									
2007	Dervin et al. [6]	Orthop	10	50	52 (47–71)	20.4 (12–46.8)	10/10 (100)	Mobile	90
2009	Krishnan and Randle [15]	JOSR	6	NR	56 (50–64)	24 (12–60)	6/6 (100)	Fixed	100
2012	Tinius et al. [31]	KSSTA	27	59.3	44 (38–53)	50 (9–71)	27/27 (100)	Fixed	100
2012	Weston-Simons et al. [34]	JBJS Br	52	21.6	51 (36–67)	60 (12–120)	51/52 (98.1)	Mobile	64.7
2015	Ventura et al. [33]	KSSTA	14	35.7	55 (45–59)	26.7 (24–40)	14/14 (100)	Fixed	100
Total	Five studies		109	36.3	50 (36–71)	48 (9–120)	108/109 (99.1)		82.4

*JBJS Br* Journal of Bone and Joint Surgery British, *JBJS Am* Journal of Bone and Joint Surgery American, *KSSTA* Knee Surgery, Sports Traumatology, Arthroscopy, *CORR* Clinical Orthopaedics and Related Research, *Orthop* Orthopaedics, *JOSR* Journal of Orthopaedics Surgery and Research, *NR* not reported

Pre-operative pain was described as the usual symptom leading to UKA in ACL-reconstructed patients, along with instability, although the latter was not clearly quantified by the authors of the papers cited.

Table 2 shows that, in patients with ACL deficiency and OA, reconstruction of the ACL was performed in patients who were significantly younger as compared to those in whom it was not performed (mean age 50 vs 66 years). This finding is likely due to the lower demand in older patients, who may cope better with instability, or indeed may be affected by a more severe pattern of arthritis, with stiffness and osteophytes contributing to stability [4].

Raw survival rates, failures, revisions, complications and re-operations per observed years are reported in Table 3. Analysing failures and revisions per 100 observed years allows a comparison across studies with different follow up periods, but it is limited by the assumption that the distribution of complications is linear over time.

**Table 3:** Survival rate data

Group	Patients followed	Mean follow up, months (range)	Raw survival rate (%)	Complications/100 observed years	Re-operations/100 observed years	Failures/100 observed years	Revisions/100 observed years
ACL-deficient UKAs	154	77 (26.4–264)	88	NR	NR	1.92	1.92
ACL-reconstructed UKAs	108	48 (9–120)	97	0.94	0.94	0.70	0.70
Total	262	65 (9–264)	92				

### Complications

According to a recently published systematic review [16], complications are considered as any deviation from the expected post-operative course, both operative and non-operative. Failures were defined as any event resulting in further surgery in which a component was changed, a new component was added or where bearing dislocation had occurred in the case of UKA [20], and any traumatic graft rupture for ACL reconstructions [30]. Any operation where the patient underwent further surgery requiring the removal and/or exchange of any material implanted during the index operation was considered as a revision.

**Fig. 7:**
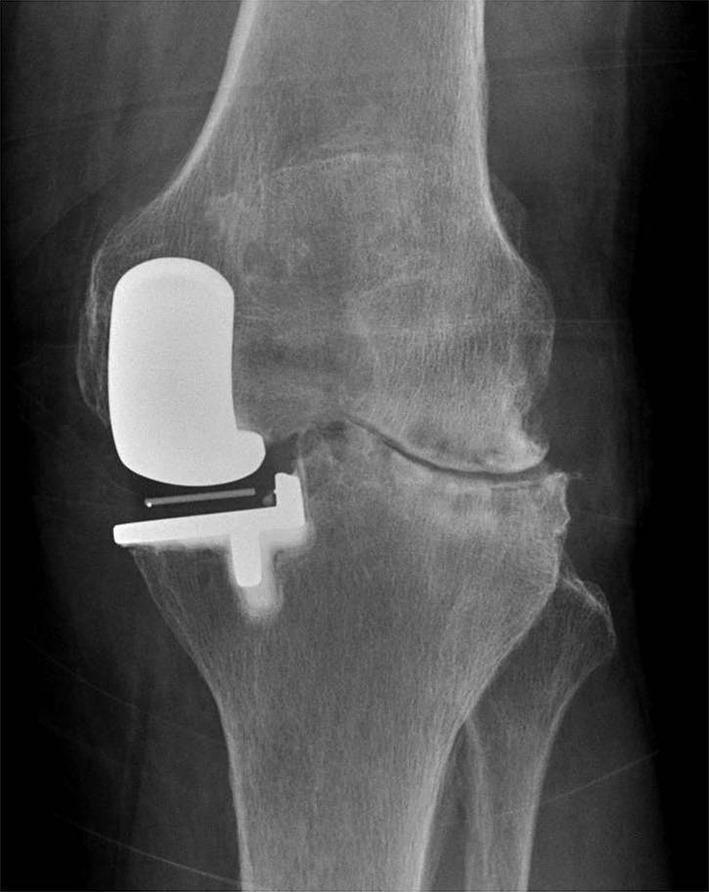
Lateral OA progression after medial UKA

UKA ACL-deficient studies showed 19 failures in 154 followed patients [12.3 %; 4 progression of lateral OA (Fig. 7), 1 painful joint replacement, 12 tibia loosening, 1 bearing instability, 1 not specified], all of which required revision (12.3 %; 10 conversions to TKA, 1 arthrodesis, 1 conversion to bi-unicompartmental arthroplasty, 7 not specified). No further complications were reported in the above mentioned group.

In UKA ACL-reconstructed papers, involving 108 patients, in addition to 3 failures (2.8 %; 1 progression of lateral OA, 1 peri-prosthetic infection, 1 bearing dislocation), all of which required revision (2.8 %; 1 conversions to TKA, 1 two-stage revision to TKA, 1 bearing substitution), 4 complications (3.7 %; 1 lateral meniscal tear, 2 stiffness, 1 loose body), managed with a re-operation (3.7 %; 3 arthroscopies, 1 manipulation under anaesthesia) were reported [16].

Patients without ACL reconstruction, compared to patients with the combined procedure, have a significantly higher failure rate (by a factor of two), with a revision rate of 1.92 % at 10 years with, as would be expected, mobile bearing UKAs having a higher revision rate than fixed bearing UKA.

Tibial component loosening is the most frequently reported reason for failure in ACL-deficient knees, especially in the oldest series from Goodfellow (Oxford, mobile bearing) and Hernigou (Lotus, fixed bearing) [9, 11]. This trend has changed in the most recent studies, which showed improved survival rates with fewer revisions per 100 observed years (1 revision per 100 observed years in recent studies vs 3.33 in the oldest series) [2, 8], with one series reporting no evidence of loosening and equivalent patient recorded outcomes to patients with an intact ACL [2].

Different clinical scores were used in the various studies, and thus it was not possible to pool them. Clinical scores data are reported in Table 4.

**Table 4:** Clinical outcomes

Year	Authors	Outcome score	Pre-op. (range)	Post op. (range)
ACL deficient UKAs				
1988	Goodfellow et al. [9]	NR	–	–
2004	Hernigou and Deschamps [11]	NR	–	–
2012	Boissonneault et al. [2]	OKS	27 (13–39)	43 (20–48)
		KSS F	70 (45–90)	100 (40–100)
		KSS O	42 (15–60)	88 (75–90)
2013	Engh and Ammeen [8]	NR	–	–
ACL-reconstructed UKAs				
2007	Dervin et al. [6]	NR	–	–
2009	Krishnan and Randle [15]	OKS	36.5 (2–40)	48
		KSS T	135 (64–167)	196 (100–200)
		WOMAC	45(35–52)	24 (21–27)
2012	Tinius et al. [31]	KSS F	38.7 (NR)	83 (NR)
		KSS O	38.4 (NR)	83 (NR)
2012	Weston-Simons et al. [34]	OKS	28 (16–46)	41 (17–48)
		KSS F	82 (45–100)	95 (45–100)
		KSS O	40 (25–80)	75 (25–95)
		Tegner	2.5 (1–5)	3.5 (1–5)
2015	Ventura et al. [33]	KOOS	62.7 (NR)	81 (NR)
		WOMAC	72.1 (NR)	85.8 (NR)
		OKS	29 (NR)	43.2 (NR)
		KSS F	80 (NR)	90 (NR)
		KSS O	45 (NR)	77 (NR)
		Tegner	2 (1–3)	3 (2–4)

*NR* Non reported, *WOMAC* Western Ontario and McMaster Universities, *OKS* Oxford Knee Score, *KSS-F and -O* Knee Society Score Functional and Objective, *KOOS* Knee injury and Osteoarthritis Outcome Score

## Conclusions

In conclusion, ACL reconstruction and UKA is our preferred treatment option for patients with ACL deficiency and bone-on-bone medial compartment arthritis, particularly in the young and active. In the elderly, isolated UKA without ACL reconstruction seems to be a reasonable and attractive option if careful patient selection is performed. The absence of clinical pre-operative instability seems to have an important prognostic role in terms of functional results, especially if ACL reconstruction is not performed with the UKA. Simultaneous or staged ACL reconstruction, although making the procedure more complex, tends to provide superior outcomes, in particular in younger and more active patients.
